# Microtubules Coordinate VEGFR2 Signaling and Sorting

**DOI:** 10.1371/journal.pone.0075833

**Published:** 2013-09-20

**Authors:** Catherine Czeisler, Takashi Mikawa

**Affiliations:** 1 Cardiovascular Research Institute, University of California San Francisco, San Francisco, California, United States of America; 2 Department of Neuropathology, The Ohio State University Medical Center, Columbus, Ohio, United States of America; Bristol Heart Institute, University of Bristol, United Kingdom

## Abstract

VEGF signaling is a key regulator of vessel formation and function. In vascular endothelial cells, this signaling is mediated through its cognate receptor VEGFR2, which is dynamically sorted in response to ligand. Little is known about the underlying mechanism of this intracellular sorting. Here we examined the role of different components of the cytoskeleton in this process. We found that VEGFR2 mainly associates with microtubule fibers and to a lesser extent with intermediate filaments and actin. Microtubule disruption leads to accumulation of VEGFR2 protein in the membrane and cytoplasm leading to defects in VEGF signaling. In contrast, inhibition of actin filaments results in no accumulation of VEGFR2 total protein or apparent changes in microtubule association. Instead, actin inhibition leads to a more global signaling disruption of the ERK1/2 pathway. This is the first report demonstrating that VEGFR2 associates closely with microtubules in modulating the subcellular sorting and signaling of VEGFR2.

## Introduction

VEGF signaling plays an important role in regulation of vasculogenesis and angiogenesis [1,2]. In vascular endothelial cells, canonical VEGF signaling involves VEGF binding to homomeric or heteromeric complexes of VEGF receptors 1 and 2 (VEGFR1 or VEGFR2) [3,4,5,6]. This results in autophosphorylation on one of several tyrosine residues in the intracellular domain of VEGFR2 [7,8]. Phosphorylation of VEGFR2 triggers a series of downstream signaling cascades as well as the uptake of the receptor complex through both endosomal and non-endosomal endocytic pathways [8,9,10,11,12].

VEGFR2 trafficking in endothelial cells has been extensively studied. In these studies, distinct pools of VEGFR2 have been documented, including a surface membrane associated pool that internalizes upon ligand binding [9] as well as an intracellular pool that is stimulated to surface upon VEGF stimulation [12]. Following activation by ligand and auto-phosphorylation, VEGFR2 either undergoes lysosomal degradation or is recycled to the membrane surface [9,10,12]. Although the various endocytic compartments trafficking VEGFR2 have been investigated, the cytoskeletal fibers that serve to route these vesicles are still unknown. Reports that microtubule inhibitors interfere with VEGF signaling in endothelial cells [13,14] point to an important role of the cytoskeleton in the signaling and dynamic sorting of VEGFR2. There are few studies to date that address the direct role of the cytoskeleton in receptor function.

VEGFR2 signaling is involved in several aspects of endothelial cell function, including differentiation, migration and survival (for a review, see [15]). Since these processes are closely associated with dynamic changes in endothelial cell shape, it is plausible that interactions between VEGFR2 and structural proteins may play a vital role in VEGFR2 subcellular sorting and signaling. In this study, we examine the interplay between cytoskeletal components and the trafficking, signaling and processing of VEGFR2 in response to VEGF.

## Results

### An intracellular pool of VEGFR2 associates with microtubules

**Figure 1:**
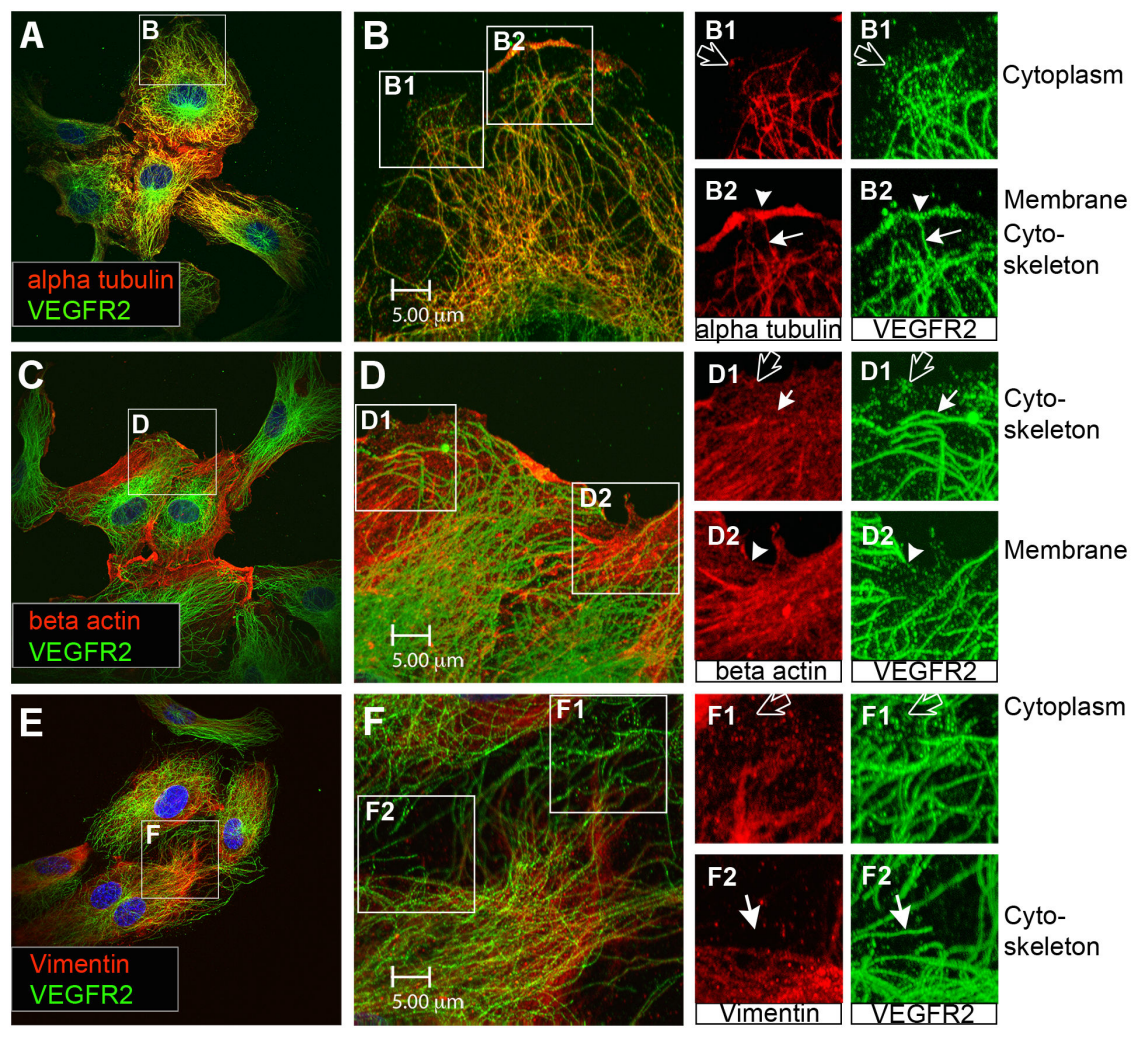
Distinct VEGFR2 pools are present in endothelial cells. (A) Endothelial cells stained for alpha tubulin (red) and total VEGFR2 (green). (B) Inset from panel A shows cell in more detail. Two insets (B1) and (B2) from panel B separate channels in red and green to more clearly show the distinct pools of VEGFR2 present in endothelial cells. This includes a pool of VEGFR2 on the cell surface (arrowhead), a granular cytoplasmic VEGFR2 pool (black arrow) and a pool of VEGFR2 arranged in fibrous arrays (white arrow). (C) Endothelial cells stained for microfilament beta actin (red) and total VEGFR2 (green). (D) Inset from panel C shows cell in more detail. The insets (D1) and (D2) from panel D separate channels into red and green to demonstrate more clearly distinct pools of VEGFR2 present in endothelial cells. This includes a pool of VEGFR2 on the cell surface (arrowhead, green), a granular cytoplasmic VEGFR2 pool (black arrow) and a pool of VEGFR2 arranged in fibrous arrays (white arrow). (E) Cell stained for intermediate filament Vimentin (red) and total VEGFR2 (green). (F) Inset from panel E shows cell in more detail. Insets (F1) and (F2) taken from panel F separate channels in green and red to show more clearly the distinct pools of VEGFR2 present in endothelial cells. This includes a granular cytoplasmic VEGFR2 pool (black arrow) and a pool of VEGFR2 arranged in fibrous arrays (white arrow). All displayed images are single focal planes.

The role of the cytoskeleton in VEGFR2 sorting and function was examined by monitoring the association between VEGFR2 and the three types of cytoskeletal fibers; microtubles, actin filaments and intermediate filaments. Immunohistochemical analysis of human aortic endothelial cells (HAECs) revealed several distinct pools of VEGFR2 including the surface membrane pool (Figure 1, arrowheads) and the cytoplasmic pool with granular appearance (Figure 1, black arrows). In addition to these well-documented VEGFR2 pools, a third pool that exhibited fibrous arrays was detected (Figure 1, white arrows). Double immunostaining of VEGFR2 (green) and cytoskeletal components (red) readily revealed overlapping of the fibrous arrays of VEGFR2 pool with alpha tubulin positive fibers (Figure 1B2 arrow). This overlap of alpha tubulin and VEGFR2 staining is demonstrated in more detail in insets B1-B2 (alpha tubulin in red and VEGFR2 in green). The fibrous arrays of VEGFR2 colocalized with tubulin fibers throughout the cell, including central regions as well as the periphery. Overlapping of VEGFR2 with beta actin (Figure 1C-D) or the intermediate filament vimentin (Figure 1E-F) was not frequently detected. The data indicate that the fibrous pool of VEGFR2 is preferentially associated with microtubules and to a lesser extent with actin and vimentin.

**Figure 2:**
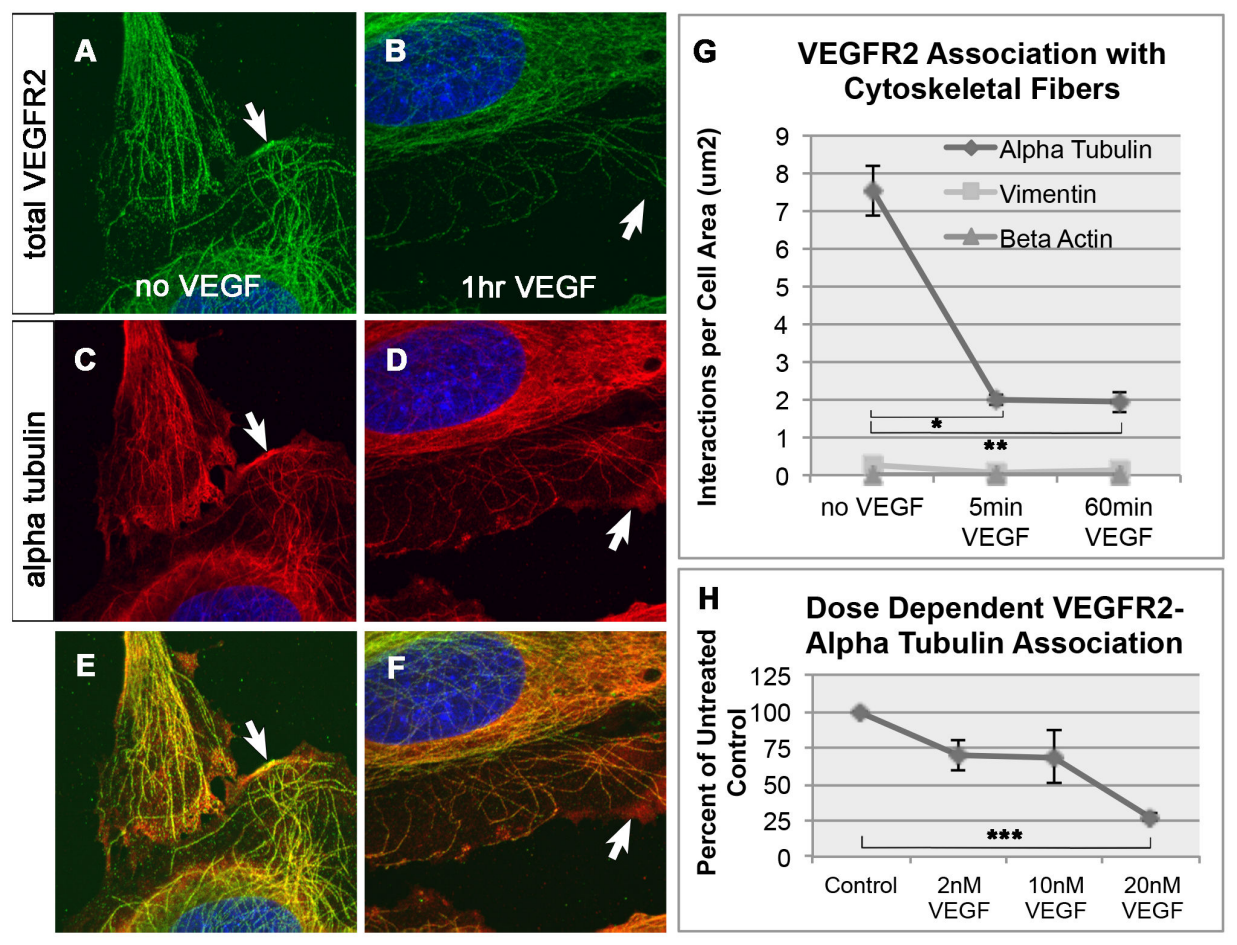
VEGF-dependent changes in VEGFR2-microtubule association. Untreated endothelial cells stained with total VEGFR2 (A), alpha tubulin (C) and merged image (E). Arrows in A, C and E denote VEGFR2 colocalizing with alpha tubulin at the cell surface. Endothelial cells stained with total VEGFR2 (B), alpha tubulin (D) and merged image (F) after treatment with 20nM VEGF for 60 minutes. Arrows in B, D and F show decreased colocalization of VEGFR2 and alpha tubulin at the cell surface. (G) Changes in the interaction between VEGFR2 and alpha tubulin, beta actin and vimentin proteins upon treatment with VEGF. Graph from one of three representative experiments is shown and bars represent standard error of the mean (SEM) within this experiment. (H) Graph showing dose response curve for VEGFR2-alpha tubulin association. This graph shows the mean result of three independent experiments normalized to untreated control. The error bars represent standard error between experiments. Significant differences (denoted by asterisks) determined using ANOVA and Tukeys post-hoc HSD test. Threshold of significance (alpha) was set to p<0.05. *p=0.0000075, **p= 0.0000073, ***p= 0.0056639.

Previous studies report changes of VEGFR2 subcellular distribution in response to VEGF, including VEGF-induced shift of VEGFR2 from early to late endosomal compartments as well as increased recycling to the membrane [9,10,12]. We therefore examined whether VEGFR2 also changed cytoskeletal association in response to VEGF. HAECs were exposed to VEGF for 60 minutes and association of VEGFR2 with microtubules was examined. A VEGF-induced decrease of a membrane associated pool was evident consistent with previous reports [10] (Figure 2A-F, arrows). A Similar VEGF-induced decrease was also detected in the fibrous VEGFR2 pool overlapping with microtubules at peripheral regions of the cell.

While the above data suggest that a VEGFR2 pool is overlapping with microtubules, the immunostaining alone do not provide high enough resolution to determine whether VEGFR2 is directly associated with microtubules. To address this issue, the VEGF-induced decline of fibrous VEGFR2 pools was analyzed by proximity ligation assay (PLA) in a quantitative manner. The PLA data showed that VEGFR2 and microtubules were localized within a distance of <40nm indicating their close association in HAECs [16]. The VEGFR2-microtubule association decreased upon addition of VEGF (Figure 2G) in a dose-dependent manner (Figure 2H). The graph shown in 2G is one representative experiment while 2H is an average of three independent experiments. This dynamic VEGF-induced change was not evident in the interaction between VEGFR2 with beta actin or vimentin. The data indicate that VEGF activation induces a specific change in the association of VEGFR2 with microtubules without detectable changes in interaction, if any, with vimentin and actin

### Microtubule disruption inhibits normal VEGFR2 subcellular distribution

**Figure 3:**
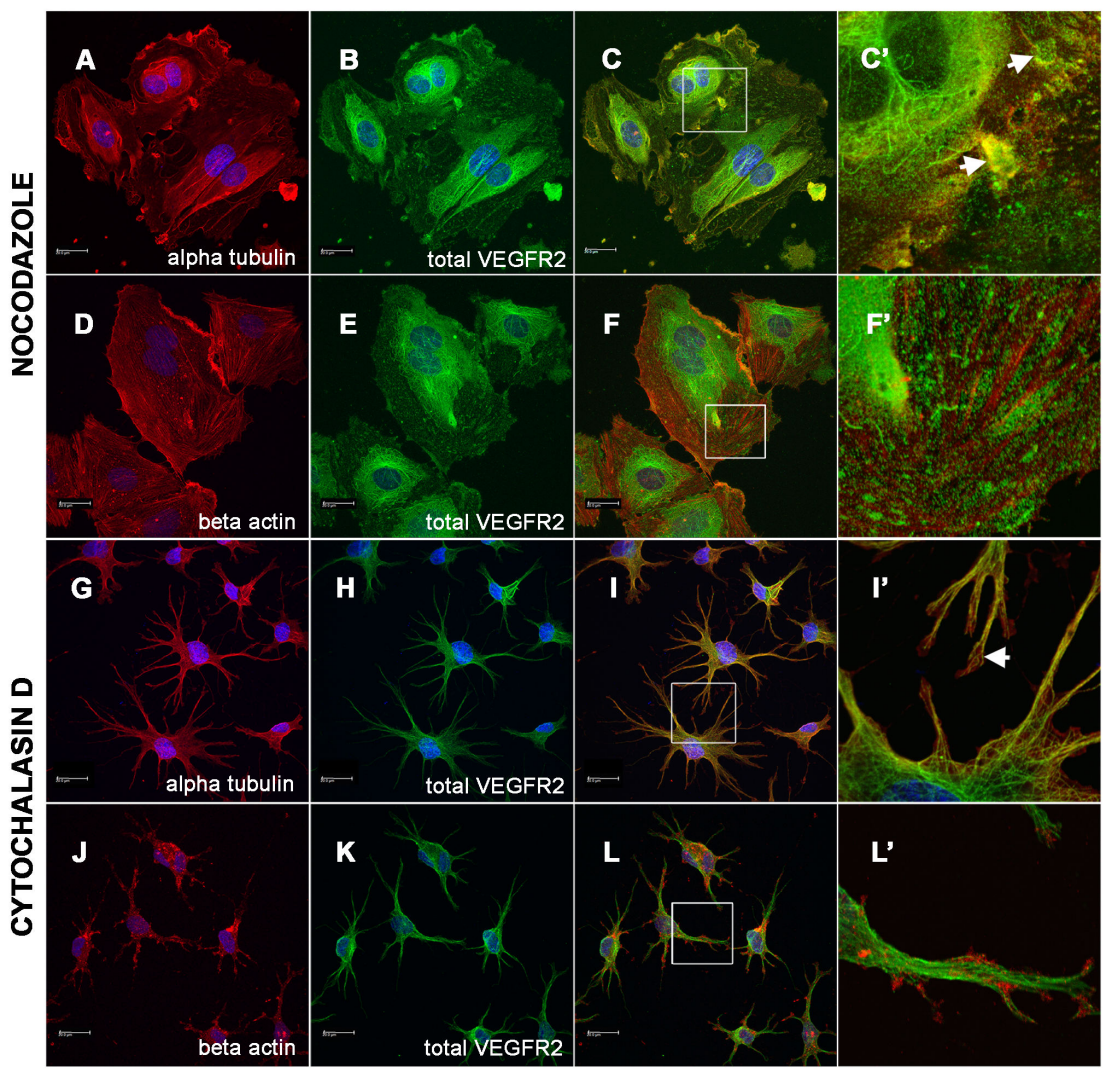
Changes in VEGFR2 subcellular localization as a result of cytoskeletal disruption. (A-F’) Endothelial cells treated for 3 hours with microtubule inhibitor Nocodazole stained with (A) alpha tubulin, (B) total VEGFR2 and (C) merged image. (C’) High power inset shows that microtubule disruption results in loss of fibrous cytoskeletal pattern of staining. VEGFR2 stain instead appears in large inclusions (arrows). (D-F’) Endothelial cells stained with (D) beta actin, (E) total VEGFR2 and (F) merged image. (F’) High power inset shows that VEGFR2 stain does not colocalize with intact actin fibers. (G-L’) Endothelial cells treated for 3 hours with actin polymerization inhibitor Cytochalasin D (CCD) and stained with (G) alpha tubulin, (H) total VEGFR2 and (I) merged image. (I’) High power image demonstrates that total VEGFR2 remains arranged in a fibrous pattern in the absence of intact actin filaments (arrow). (J) Endothelial cells stained with beta actin, (K) total VEGFR2 and (L) merged image. (L’) High power image shows that CCD treatment results in accumulation of beta actin at the periphery of processes and that total VEGFR2 remains organized in a fibrous cytoskeletal pattern despite the disruption of actin filaments.

To test whether the association with microtubules is necessary for normal VEGFR2 trafficking, we disrupted microtubules by treatment of endothelial cells with Nocodazole. The Nocodazole treated HAECs showed a decrease in the appearance of intact microtubule fibers (Figure 3A), indicating effective disruption of microtubules. The Nocodazole treated cells also displayed decreased fibrous VEGFR2 arrays and a complimentary increase in granular VEGFR2 staining (Figure 3C’ arrows). This aberrant VEGFR2 distribution was specific to microtubule disruption since cells treated with the actin polymerization inhibitor Cytochalasin D (CCD) did not show any apparent changes in subcellular distribution of fibrous VEGFR2 (Figure 3G-L’). CCD treated cells similar to untreated cells, showed a bead-like VEGFR2 staining pattern along microtubule fibers (Figure 3I’ arrows). These data suggest that the observed steady-state fibrous subcellular distribution of VEGFR2 is dependent on the presence of intact microtubules.

### Microtubule disruption inhibits normal VEGFR2 signaling

**Figure 4:**
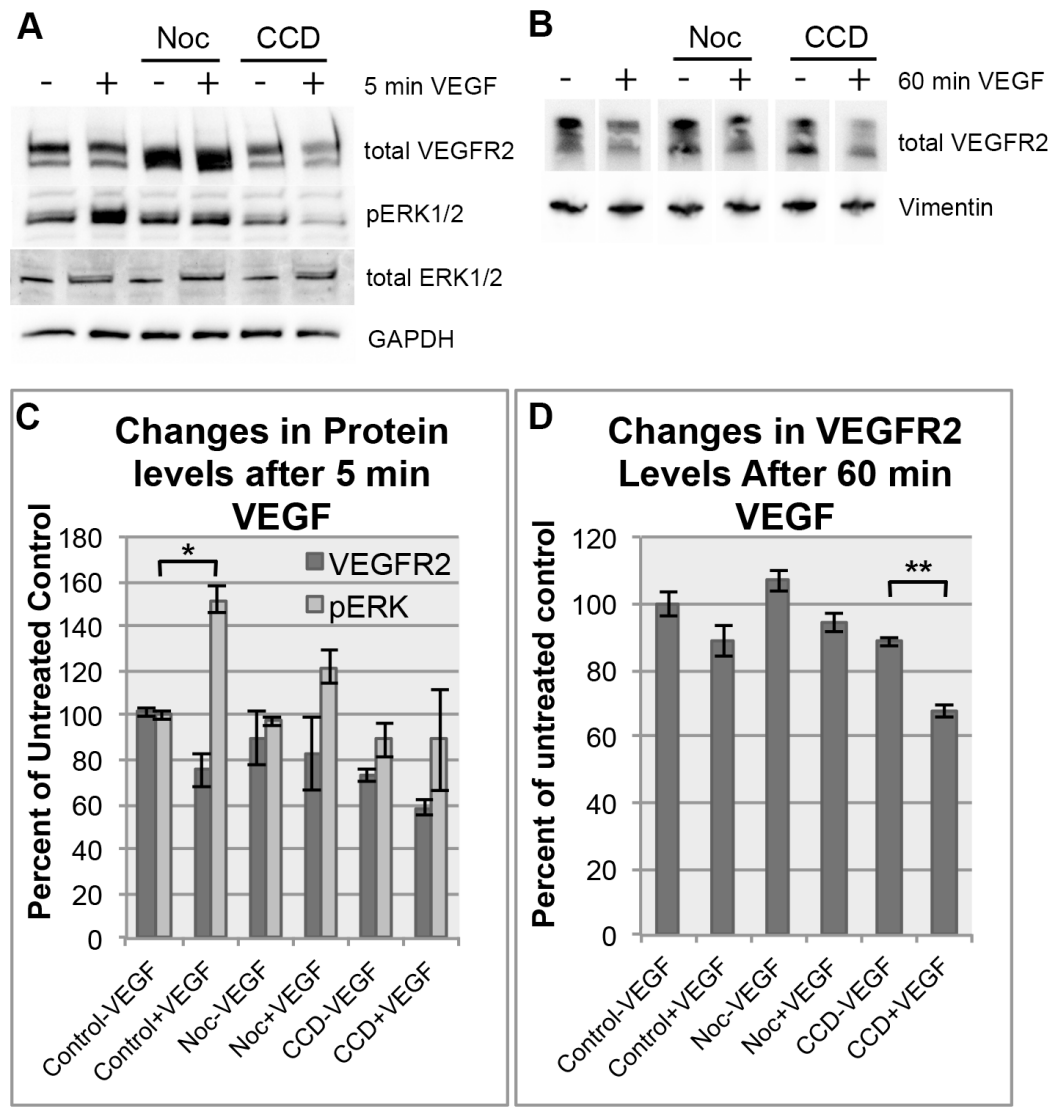
Cytoskeletal disruption leads to a delay in the VEGFR2 response. (A) Endothelial cells treated with cytoskeletal inhibitors Nocodazole or CCD show a loss of immediate ERK1/2 phosphorylation (pERK1/2) after 5 minutes of VEGF stimulation compared to controls while total ERK1/2 levels remain constant. (B) Endothelial cells treated with cytoskeletal inhibitors, similar to controls, undergo a decrease in the total VEGFR2 levels after 60 minutes of VEGF stimulation. Panels (C) and (D) are data derived from 2 independent experiments and show the mean densitometry of these bands normalized to untreated control levels. Significant differences (denoted by asterisks) determined using paired student’s T-Test. Threshold of significance (alpha) was set to p<0.01. *p= 0.00014, **p= 0.00049.

The above imunofluorescence and PLA data suggested that microtubules were the major component responsible for the fibrous cytoskeletal pool of VEGFR2. These data raise the question of whether microtubules are also involved in the signaling mediated by this receptor. This possibility was tested by biochemical methods independent of VEGFR2 antibody-based imaging analysis. Upon VEGF stimulation, VEGFR2 is phosphorylated at several tyrosine residues on its C-terminus [6]. We examined whether microtubules regulate this early signaling step. The molecular disruption of microtubules by Nocodazole did not induce a detectable decline of total VEGFR2 levels but resulted in a decrease in VEGF induced phosphorylation of second messenger pERK1/2 levels compared to control. In contrast to Nocodazole, actin inhibitor, Cytochalacin D (CCD), decreased levels of total VEGFR2. As a result, pERK1/2 signals displayed below baseline levels (Figure 4A and C). The decrease in total VEGFR2 protein as a result of actin disruption, particularly upon VEGF treatment indicates a broader inhibition by CCD treatment distinct from Nocodazole effects on VEGFR2 signaling.

In addition to the above phosphorylation reactions which are an acute response to VEGF, a decline of VEGFR2 protein levels is a well documented slow reaction to VEGF treatment [10]. Consistent with this notion, VEGF-treated HAECs decreased VEGFR2 levels in control conditions. Nocodazole-treated cells also exhibited a consistent decrease of total VEGFR2 protein upon VEGF treatment (Figure 4B and D). CCD-treated cells exhibited higher variations in VEGF induced changes of VEGFR2 protein levels. It is important to note that baseline VEGFR2 levels vary between control, Nocodazole and CCD-treated groups. In particular, decreased levels of VEGFR2 were evident in the CCD treated condition compared to control. These data suggest distinct roles for microtubules and actin in the regulation of VEGFR2 levels and receptor-mediated VEGF signaling events.

### Microtubule inhibition disrupts normal subcellular sorting of VEGFR2

**Figure 5:**
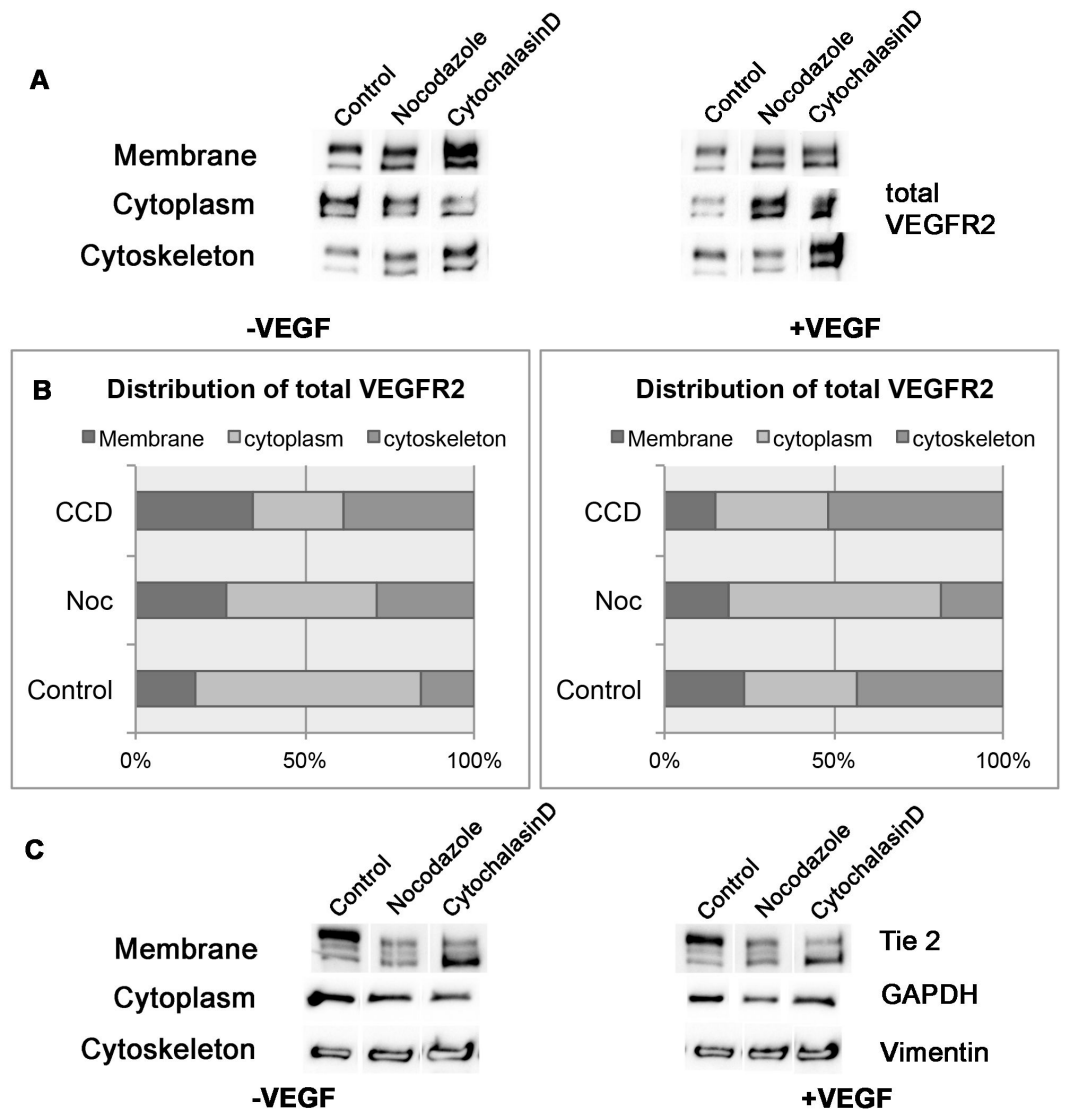
Cytoskeletal involvement in VEGF-dependent subcellular compartment change. (A) Treatment of endothelial cells with VEGF and/or cytoskeletal inhibitors and subsequent subcellular fractionation into membrane, cytoplasmic and cytoskeletal fractions (top to bottom row, in order). (B) Densitometry plots of (A) showing differences in distribution of total VEGFR2 with and without VEGF treatment among the three subcellular compartments analyzed. Data is represented as a percent of total VEGFR2 levels for each VEGF condition. (C) Loading controls used for each subcellular fraction.

VEGFR2 levels within the cell are regulated in part by VEGF-dependent receptor uptake from the membrane and subsequent sorting into subcellular compartments. While the above experiments showed that microtubule disruption increased basal VEGFR2 levels in the cell, it remains unclear whether microtubules played a role in the VEGF-dependent sorting of VEGFR2 within the cell. To test this possibility, VEGF-induced changes in subcellular distribution of VEGFR2 were analyzed in the presence or absence of microtubule disruption. Proteins extracted from the membrane, cytoskeletal and cytoplasmic compartments were collected and analyzed by Western blot (Figure S1). Upon VEGF addition, control HAECs exhibited a decrease in membrane and cytoplasmic VEGFR2 pools and a slight increase in the cytoskeletal pool. In striking contrast, the Nocodazole-induced disruption of microtubules resulted in a decrease in the cytoskeletal pool and preferential accumulation of total VEGFR2 in the membrane and cytoplasmic fractions (Figure 5). Actin disruption by CCD resulted in the accumulation of membrane and cytoskeletal VEGFR2. CCD treated cells showed a very different distribution of VEGFR2 after VEGF stimulation. Most of the VEGFR2 accumulated in the cytoskeletal fraction, presumably associated with intact microtubules. The result is consistent with the above immunofluorescence and PLA data suggesting that microtubules play a role in maintaining steady state cytoskeletal and membrane bound receptor distribution. The data support the model that upon stimulation, VEGFR2 is trafficked from the membrane to the intracellular space via microtubules. CCD-treated cells still show a bead-like pattern of VEGFR2 stain along microtubules. These results are also consistent with the above findings that VEGFR2 binds more prominently to microtubules than actin, although the data do not rule out the interaction between intact microtubules and actin that could be important for maintenance of steady state VEGFR2 distribution. Taken together, the data indicate that the microtubule cytoskeleton is the major component regulating VEGFR2 subcellular localization.

## Discussion

**Figure 6:**
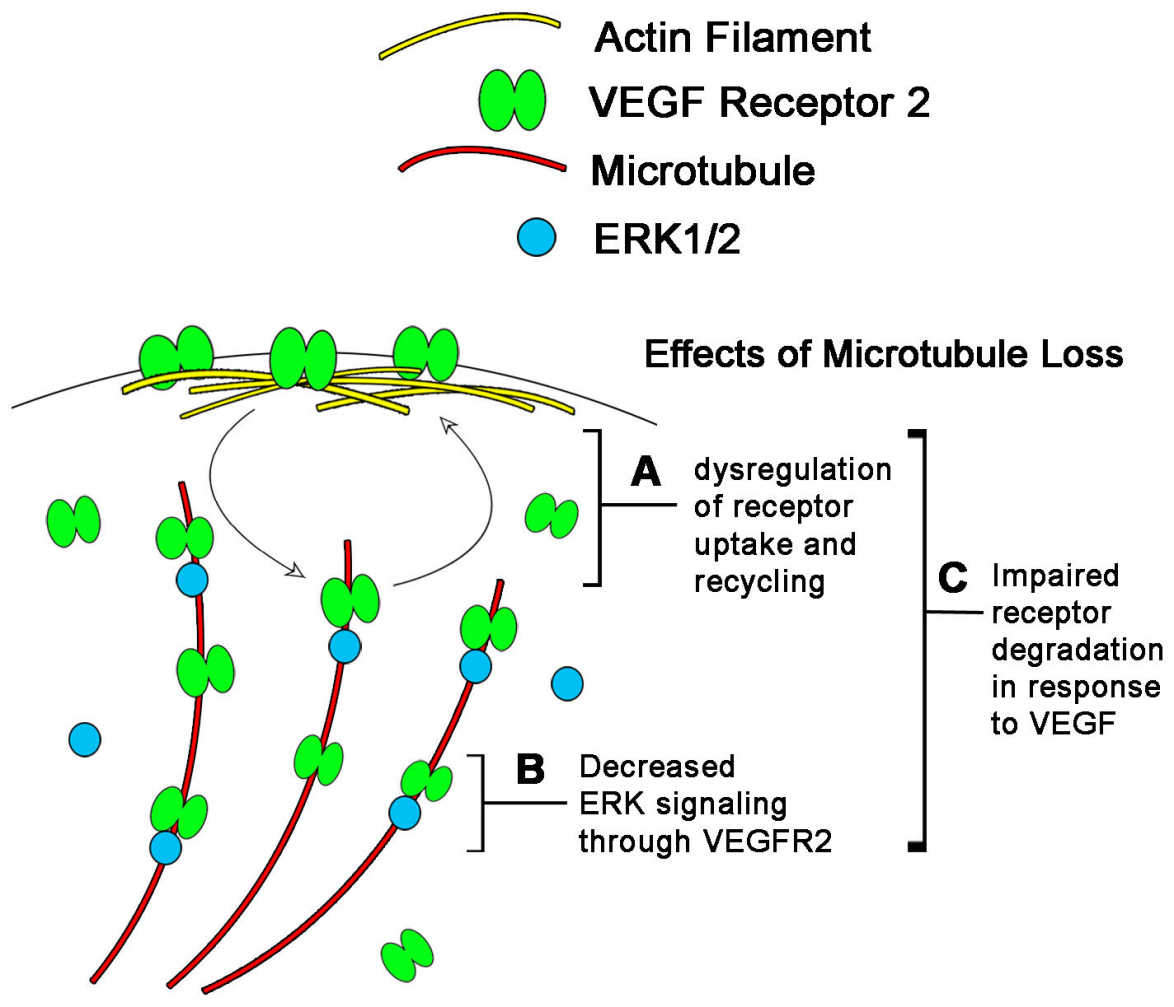
Proposed Model of Microtubule Role in VEGFR2 Signaling. This model is based on the findings presented in this study. VEGFR2 exists in an equilibrium concentration on endothelial cell membranes due to its constant internalization and recycling. (A) The disruption of microtubules causes this equilibrium membrane concentration to increase indicating that it is either interfering with uptake or recycling of the receptor to the membrane. In response to VEGF, VEGFR2 becomes phosphorylated, internalizes and activates second messenger molecules such as ERK1/2. (B) We propose that VEGFR2 is trafficked along microtubules after VEGF activation thus increasing the likelihood that it will come in contact with MAP Kinases such as ERK1/2 that are known to associate with the microtubule cytoskeleton. In the absence of microtubules, the immediate phosphorylation of ERK1/2 is much less efficient. Finally, the total VEGFR2 amounts in the cell are dictated by the rate of degradation of the receptor. (C) Since actin and microtubule disruption changes total levels of VEGFR2, it is likely that the cytoskeleton also plays a function in increasing the efficiency of receptor degradation in response to VEGF.

VEGF-induced dynamic trafficking of VEGFR2 has been extensively studied, but the underlying intracellular sorting mechanism for VEGFR2 remains poorly understood. The present study has identified a novel role for the cytoskeletal fibers that direct the flow of VEGFR2 in response to ligand. Our data are consistent with a model (Figure 6) where VEGF-induced VEGFR2 subcellular sorting and signaling is at least in part mediated by a close specific association between microtubules and VEGFR2, which is not prominent with actin or intermediate filaments.

The above model is supported by our immunofluorescence data that have identified for the first time a subcellular pool of VEGFR2 along a cytoskeletal network in addition to the previously reported membrane-bound and cytoplasmic pools. Although many antibodies for VEGFR2 have been available, the presence of a cytoskeletal pool has not been well documented. Our survey of the four most commonly used antibodies in the literature has revealed two staining patterns (Figure S2): one displays predominately punctate and perinuclear staining with a less fibrous pattern (Figure S2 A, B and C) and the other exhibits a punctate pattern and a distinct fibrous pattern (Figure S2 B inset and D). It is currently unclear how these antibodies recognize distinct pools of VEGFR2 at various extents. However, our direct subcellular fractionation has detected a significant portion of VEGFR2 in a cytoskeletal fraction.

The present study provides biochemical evidence for a distinct pool of VEGFR2 associated with a cytoskeletal network. This idea is also consistent with the well documented subcellular trafficking of VEGFR2 upon binding to VEGF.

Our data suggest that microtubules are the major cytoskeletal component along which VEGFR2 is associated. Our imaging data show co-localization of VEGFR2 along microtubules but less so along actin or intermediate filaments. Since the overlapping fluorescence signal alone does not provide high enough resolution to prove a direct association between VEGFR2 and microtubules, we used proximity ligation assays (PLA) in order to verify this association. Our PLA results successfully demonstrated that these two proteins are within interacting distance in situ. PLA data also revealed lower levels of interactions between VEGFR2 and either actin or intermediate filaments. Based on these data, it is likely that association of VEGFR2 with microtubules is more prominent than with other cytoskeletal components. Our finding that disruption of microtubule networks yielded severe impairment in protein subcellular localization further demonstrated a critical role for microtubules in the proper intracellular sorting of VEGFR2.

Supporting this model where microtubules play an active role in sorting and trafficking of VEGFR2, our data demonstrate that microtubule fibers also regulate VEGFR2 receptor signaling after VEGF stimulation. This regulatory function was evident when Nocodazole treated cells failed to phosphorylate ERK1/2 Map kinase even in the presence of VEGF. It is currently unclear how the absence of microtubules inhibits the ability of VEGFR2 to phosphorylate second messengers in response to ligand. It is possible that Nocodazole disrupts subcellular localization of VEGFR2 in such a manner that it becomes no longer accessible to ligand. However, subcellular fractionation studies show that in Nocodazole-treated HAECs there is still VEGFR2 on the membrane surface and this pool of receptor is able to respond to VEGF by phosphorylating and internalizing after prolonged treatment. As such it is unlikely that the receptor is simply not binding to ligand due to mislocalization. Both the lack of immediate ERK1/2 phosphorylation and the presence of a delayed response to VEGF suggest that the association between VEGFR2 and microtubules plays a role in establishing the correct timing of receptor activation after ligand stimulation.

Consistent with this idea, our data demonstrate that microtubules coordinate ligand-mediated trafficking of VEGFR2. Subcellular fractionation data shows that microtubule disruption causes membrane accumulation of VEGFR2. This is consistent with an idea that microtubules play a role in either the uptake or the recycling of receptor to the membrane (Figure 6A). Our subcellular fractionation experiments also show that VEGF treatment elicits a net flux of VEGFR2 from the membrane and cytoplasm to the cytoskeleton. In the absence of intact microtubules, all the membrane bound VEGFR2 instead accumulates in the cytoplasm. This is consistent with a model where VEGFR2 is being trafficked preferentially along microtubules after exiting the membrane. Microtubules in this instance may serve as a scaffold to bring VEGFR2 and second messenger molecules in closer proximity to increase activation (Figure 6B). This model is supported by our data showing that the pERK1/2 response is diminished after microtubule disruption. This role in ligand-mediated trafficking appears to be unique to microtubules since actin disruption does not yield similar effects in VEGFR2 subcellular localization. Subcellular fractionation and immunofluorescence data demonstrate that in the absence of intact actin filaments, VEGFR2 remains associated with the cytoskeleton. The present study also supports a model where microtubules regulate VEGFR2 intracellular sorting in response to ligand. It is likely that this intracellular sorting involves the degradation of receptor in response to ligand. Our data show that loss of microtubules leads to an increase in total VEGFR2 levels. Thus, it is plausible that microtubules also play a role in VEGF-mediated receptor degradation (Figure 6C). The exact mechanism as to how microtubules regulate VEGFR2 functions remains to be determined in the future. Identification of motor protein(s) responsible for the microtubule-mediated intracellular trafficking would further facilitate our understanding of the VEGFR2 signaling network.

The present study provides cell biological and biochemical evidence for a novel regulatory role of microtubules in the trafficking and signaling of VEGFR2. Understanding the nature of this regulation could provide insight into VEGF-signaling mechanisms in both health and disease, including vascular development diseases as well as cancers resulting from disregulated expression of this receptor.

## Materials and Methods

### Cell culture

Human Aortic Endothelial Cells were purchased from Lonza (CC-2535) and propagated with the EGM-2 BulletKit (Lonza CC-3162) at a density of 7,500 cells/cm^2^ according to the manufacturer’s instructions.

### Immunofluorescence

Cells were grown in 24-well plates on glass coverslips until 70% confluent. Cells were then fixed with 4% Paraformaldehyde for 15 minutes at room temperature, followed by ice-cold 100% Methanol for 10 minutes. Cells were washed with PBS and incubated with VEGFR2 antibody (Abcam ab-9530, lot GR71669-4), Vimentin antibody (Cell Signaling Technology #5741), beta actin antibody (Cell Signaling Technology #8457) and alpha tubulin antibody (Abcam ab18251). All primary antibody incubations were performed in PBS containing 5% Normal Goat Serum and 0.01% Triton X-100 at 4°C overnight. Alexafluor-488 and 594 conjugated antibodies were used as secondary antibodies for detection (Invitrogen). DAPI was used as a nuclear contrast dye.

### Chemical/Cytokine treatments

Where indicated, HAECs were treated with cytoskeletal inhibitors Nocodazole (0.5µM) and Cytochalasin D (2µM) (Sigma) for 3 hours prior to fixation for immunofluorescence or protein extraction for Western blot analysis. HAECs were treated for 5 minutes, 30 minutes or 60 minutes with 20nM VEGF_165_ (V7259 Sigma). All treatments were performed in growth media (EGM) without prior serum starvation.

### Proximity ligation assay (PLA)

The same protocol used for the immunofluorescence was followed for detection of protein. Instead of secondary antibody for detection, the Duolink ^®^II kit was used to perform the hybridization, ligation and PCR for detection of interacting proteins (Olink Bioscience, Uppsala, Sweden) according to manufacturer’s instructions.

### Subcellular fractionation

Endothelial cells were grown to 60-70% confluence in T-500 dishes. Subcellular proteins were extracted using the Compartmental Protein Extraction Kit (215RF Millipore, Temecula, CA) according to manufacturer’s protocol. Briefly, this process first utilizes shear stress to lyse cells without perturbing nuclei. Supernatants resulting from this step contain soluble cytoplasmic proteins. Subsequent steps isolate membrane proteins and cytoskeletal proteins by a series of differential detergent extractions. The detergents contained in the extraction buffers were incompatible with traditional BCA assays for protein quantitation. Therefore, proteins were corrected for loading by performing western blots using compartment-specific antibodies and reloading samples so that these stains were comparable between lanes.

### Western blot analysis

Whole protein lysate was extracted from 60-70% confluent endothelial cells by aspirating media, washing once with PBS and adding lysis buffer containing 1% SDS, 1mM EDTA, 5% glycerol and 1X Halt ^®^Protease/Phosphatase inhibitor (Pierce). Samples were scraped from the dish, triturated through a 28-gauge needle several times and incubated at 95°C for 5 minutes. Samples were diluted in Laemmli buffer containing DTT and incubated at 95°C for 5 minutes. SDS-PAGE was performed using Mini-Protean 3 gel running system and precast 4-20% Tris-Glycine gel (Biorad). Proteins were transferred onto nitrocellulose membranes and blocked using TBS containing 0.01% Tween and 5% nonfat milk for 30 minutes. Membranes were incubated with primary antibodies to total VEGFR2 (Cell Signaling Technology #2479), pY1214-VEGFR2 (Invitrogen #441052), pERK1/2 (Abcam ab50011), Vimentin (Cell Signaling Technology #5741), Histone H1 (Santa Cruz Biotechnology sc-8030) and GAPDH (Millipore MAB374). Primary antibody incubations were performed overnight at 4°C. Membranes were washed with TBS-T and incubated with anti-mouse or anti-rabbit HRP-conjugated secondary antibodies (Santa Cruz Biotechnology) for 1 hour at room temperature and detected with Immun-Star^TM^ WesternC^TM^ Kit (Biorad #170-5070) using a Molecular Imager^®^ ChemiDoc ^TM^XRS+ with ImageLab^TM^ Software (Biorad).

### Microscopy

All confocal images were captured using a LeicaTCS SPEII confocal microscope with Leica Application Suite Advance Fluorescence software version 2.4.1. Z-stacks were set up to scan a 10µm depth using a step size of 0.17µm.

### Quantification of results

Densitometry of Western blot bands was performed using Adobe Photoshop CS6 (Adobe). Briefly, the image was opened in 8-bit Tiff format and the image was inverted. Protein bands were selected using the rectangular selection tool and the “mean” number value in the histogram was recorded. Bands were measured using the same size rectangle in all western blots. This mean was normalized to the experiment’s own loading control. The resulting normalized results were averaged across experiments and plotted as bar graphs in figures. Significant differences between conditions within this same experiment were determined using students paired T-Test and denoted graphically by asterisks. Our threshold of significance (alpha) was set to p<0.01.

The experiments in Figure 2 were repeated 3 times with similar results. The graph in Figure 2G is one representative experiment. PLA results for Figure 2G were quantified using ImageJ in the following manner. The confocal z-stack was converted into 8-bit format and opened by ImageJ. The z-stack was then thresholded in ImageJ by assigning an upper and lower threshold to separate particles for counting. The same upper and lower thresholds were maintained for the entire series of images in a single experiment. The number of PLA interactions (dots) was counted throughout the z-stack using the “analyze particles” function in ImageJ. The resulting number was divided by the total cell area within an image to generate the number of interactions per cell area. Significant differences between conditions within this same experiment were determined using ANOVA and Tukeys post-hoc HSD test (R v2.12) and denoted graphically by asterisks. Our threshold of significance (alpha) was set to p<0.05.

Figure 2H represents the mean values from three independent experiments. Briefly, PLA per cell area values were calculated as outlined above. These values were expressed as a percentage of the untreated control within the same experiment. These normalized values were then averaged across the three experiments to generate the dose-response graph. The error bars represent the standard error across experiments. Significant differences between VEGF dosages across experiments were determined as above using ANOVA and Tukeys post-hoc HSD test (R v2.12) setting alpha at p<0.05.

## Supporting Information

Figure S1 **Survey of commonly used VEGFR2 antibodies.** Immunofluorescence of endothelial cells using (A) Santa Cruz monoclonal mouse antibody sc-6251, (B) Cell Signaling monoclonal rabbit antibody 2479 lot 18 and lot 10 (inset), (C) R&D goat polyclonal antibody AF357 and (D) Abcam mouse monoclonal antibody ab9530. Antibodies were tested in western blot (E) on proteins extracted from human embryonic kidney cells (HEKs), human aortic endothelial cells (HAECs) and human embryonic kidney cells transfected with VEGFR2 (HEK+VEGFR2). GAPDH was used as a loading control in panel E. Panel E western blots are labeled with the molecular weights of the bands recognized by each antibody. Fully glycosylated VEGFR2 migrates at approximately 230kDa. Partially glycosylated or immature VEGFR2 migrates at approximately 200kDa. Unglycosylated VEGFR2 migrates at approximately 150kDa.
(TIF)

Figure S2 **Subcellular fractionation of endothelial cells.** Subcellular fractions were extracted from cultured endothelial cells and lysates were analyzed by Western blot. Extracts were tested for enrichment by blotting for compartment specific antibodies as follows: Tie2 antibody for membrane proteins (first row), GAPDH antibody for cytoplasmic proteins (second row), Vimentin antibody for cytoskeletal proteins (third row) and Histone H1 antibody for nuclear proteins (fourth row).
(TIF)
