# Supplementary figures and images for: Microtubules Coordinate VEGFR2 Signaling and Sorting
Source: PLoS One. 2013 Sep 20;8(9):e75833. doi: 10.1371/journal.pone.0075833 (PMC3779169; doi:10.1371/journal.pone.0075833)

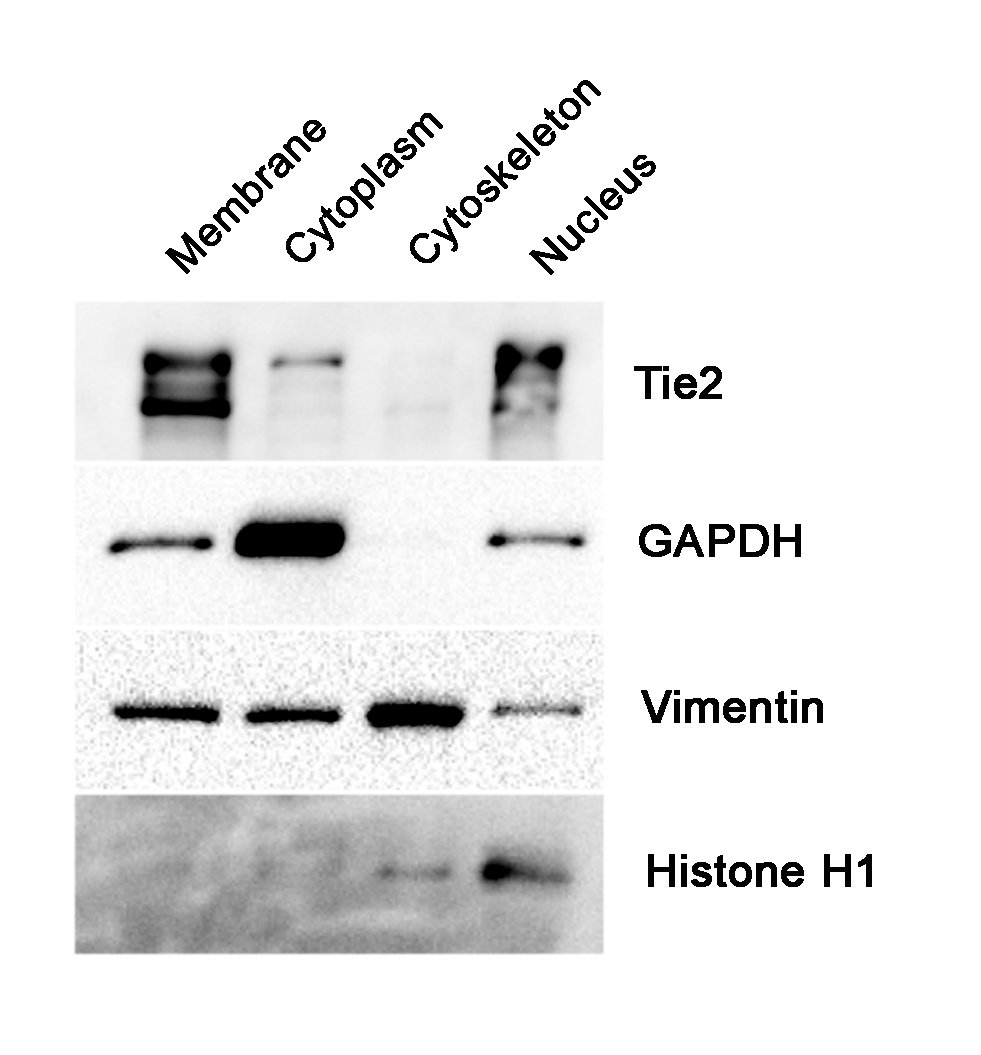

Supplement: Figure S1 — Survey of commonly used VEGFR2 antibodies. Immunofluorescence of endothelial cells using (A) Santa Cruz monoclonal mouse antibody sc-6251, (B) Cell Signaling monoclonal rabbit antibody 2479 lot 18 and lot 10 (inset), (C) R&D goat polyclonal antibody AF357 and (D) Abcam mouse monoclonal antibody ab9530. Antibodies were tested in western blot (E) on proteins extracted from human embryonic kidney cells (HEKs), human aortic endothelial cells (HAECs) and human embryonic kidney cells transfected with VEGFR2 (HEK+VEGFR2). GAPDH was used as a loading control in panel E. Panel E western blots are labeled with the molecular weights of the bands recognized by each antibody. Fully glycosylated VEGFR2 migrates at approximately 230kDa. Partially glycosylated or immature VEGFR2 migrates at approximately 200kDa. Unglycosylated VEGFR2 migrates at approximately 150kDa. (TIF) [file pone.0075833.s001.tif]

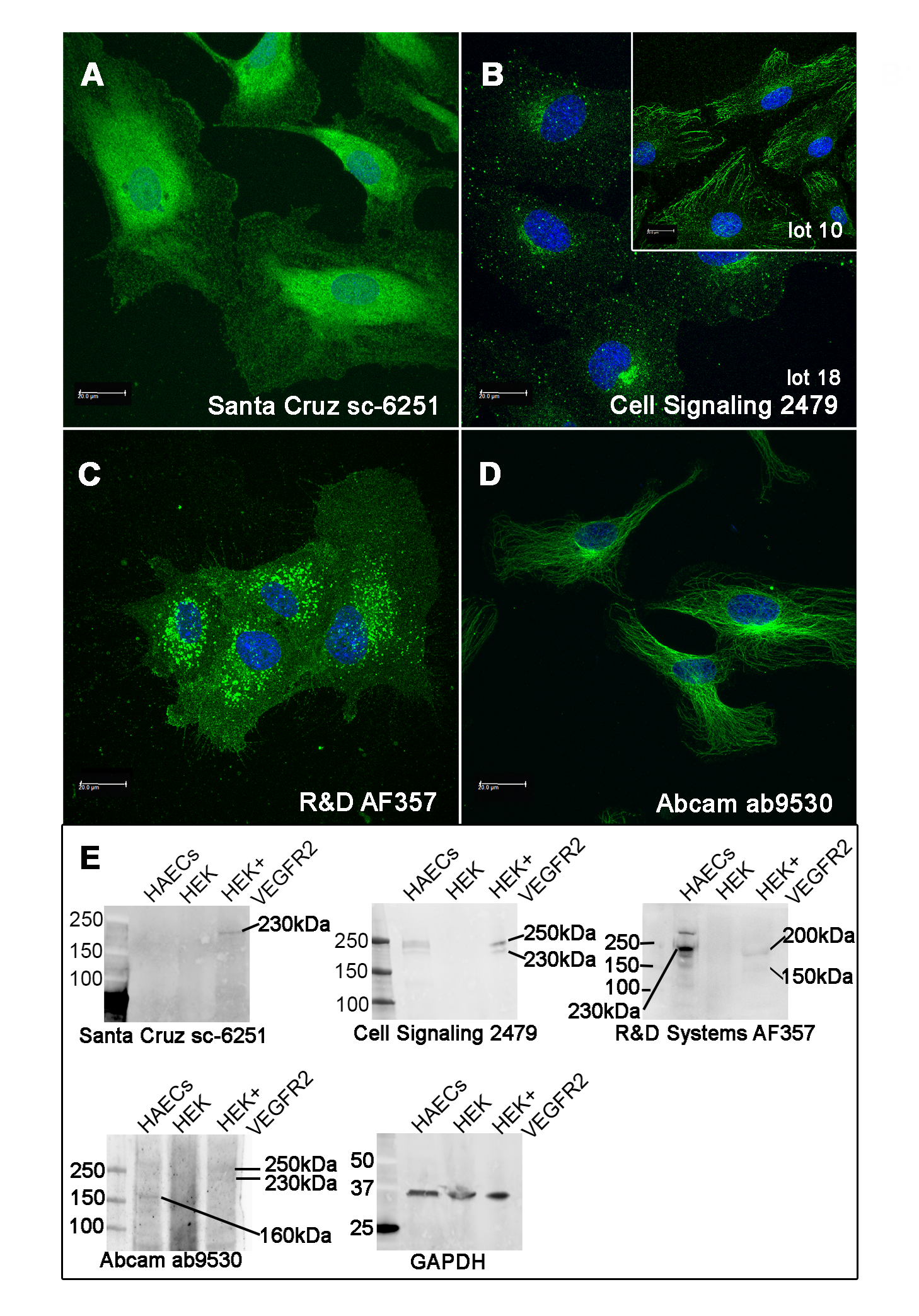

Supplement: Figure S2 — Subcellular fractionation of endothelial cells. Subcellular fractions were extracted from cultured endothelial cells and lysates were analyzed by Western blot. Extracts were tested for enrichment by blotting for compartment specific antibodies as follows: Tie2 antibody for membrane proteins (first row), GAPDH antibody for cytoplasmic proteins (second row), Vimentin antibody for cytoskeletal proteins (third row) and Histone H1 antibody for nuclear proteins (fourth row). (TIF) [file pone.0075833.s002.tif]
